# Trophic and Microbial Dynamics in a Mediterranean Transitional Ecosystem (Lake Faro, Southern Italy): Implications for *Pinna nobilis* Conservation

**DOI:** 10.3390/microorganisms14020423

**Published:** 2026-02-11

**Authors:** Gabriella Caruso, Salvatore Giacobbe, Filippo Azzaro, Franco Decembrini, Marcella Leonardi, Giovanna Maimone, Adriana Profeta, Paola Rinelli

**Affiliations:** 1 Institute of Polar Sciences (ISP), National Research Council (CNR), Section of Messina, Spianata S. Raineri 86, 98122 Messina, Italy; filippo.azzaro@cnr.it (F.A.); franco.decembrini@cnr.it (F.D.); marcella.leonardi@cnr.it (M.L.); giovanna.maimone@cnr.it (G.M.); 2 Institute of Biological Resources and Marine Biotechnologies (IRBIM), National Research Council (CNR), Spianata S. Raineri 86, 98122 Messina, Italy; salvatore.giacobbe@unime.it (S.G.); adriana.profeta@cnr.it (A.P.); paola.rinelli@cnr.it (P.R.)

**Keywords:** water quality, trophic status, biogeochemical processes, *Pinna nobilis*, transitional environments

## Abstract

Transitional water bodies are coastal areas of strategic naturalistic and socio-economic importance, and highly vulnerable to increased anthropic pressure. A monitoring study was performed in the transitional area of Lake Faro (Cape Peloro Lagoon, Italy), where specimens of the threatened species *Pinna nobilis* (Mollusca, Bivalvia) constitute a resident community, as a part of a wider research program aiming to preserve this organism in the context of safeguarding lake biodiversity. Five surface water samplings with a two-month frequency were carried out at four selected stations, three of which were located in the inner part of the lake and one control station outside, in a canal connecting the lake to the Messina Straits. Trophic conditions (total suspender matter, nutrients and chlorophyll-a) and the main environmental variables (temperature, salinity, dissolved oxygen) were measured. Insights into the total prokaryotic abundance and metabolism via the extracellular enzymatic activities (i.e., leucine aminopeptidase; beta-glucosidase and alkaline phosphatase) were obtained. The dataset indicated that microbial and trophic dynamics were associated with the abundance of the *P. nobilis* population. These parameters, moreover, proved to represent a suitable tool for characterizing the environmental health status of transitional areas, as well as for implementing new effective strategies for sustainable resource management.

## 1. Introduction

Coastal transitional environments, such as lagoons, estuaries, salt marshes, beaches and tidal flats act as interfaces between terrestrial and marine ecosystems and are highly dynamic systems due to tides, salinity gradients, and organic matter inputs [1]. Within the coastal zone, these areas play a naturalistic value, as they are biodiversity hotspots hosting migratory fauna, acting as a nursery for numerous species, and favoring the settlement of ecologically relevant species due to the availability of sheltered habitats. However, their health status is very vulnerable to the impact of human pressure from urban settlements, together with seasonal pressures related to ecosystem services like tourism and recreational activities. Excessive organic load may alter the normal biogeochemical fluxes and cause outbreaks of eutrophication phenomena including dystrophic and anoxic crises, due to enhanced dissolved oxygen consumption [2]. These events are generally associated with a reduction in the diversity of the biological communities hosted in these ecosystems, with mostly unknown consequences for human activities and health. The multidisciplinary and integrated study of transitional ecosystems responds to the growing demand for scientific and technological information necessary for the sustainable management of the coastal zone and, therefore, for the preservation of these fragile habitats and their associated resources. Sound environmental management policy for aquatic resources cannot be separated from a deep knowledge of environmental conditions and the trophic dynamics that regulate the ecosystem functioning.

Functional characterization of natural ecosystems relies on knowledge of components, processes, and structural relationships. Poor functionality in the lower trophic levels inevitably results in ecosystem disequilibrium, affecting secondary and tertiary consumers in terms of both productivity and species richness. Microbial decomposition in transitional environments represents a key ecological process, allowing the natural breakdown of organic polymers into simpler monomers, and the regeneration of carbon and nutrients in their inorganic forms [3].

Shallow aquatic environments receive a complex and diversified range of organic matter including marine detritus, freshwater load inputs, plant litter, phytoplankton and anthropogenic waters. Among microbial communities, heterotrophic bacteria (i.e., Proteobacteria, Bacteroidetes) are major players in most decomposition processes; in addition, fungi contribute particularly to plant litter decomposition, while Archaea play a role under anoxic conditions. While microbial communities play an important role in the decomposition of both particulate (POM) and dissolved (DOM) organic matter and in nutrient cycling, organic matter inputs in turns affect microbial community structure and function [4].

Effectiveness of the integrated study of trophodynamics and microbial metabolism in assessing the environmental quality of Sicilian transitional areas was shown [1,5,6]. In Cape Peloro Lagoon ecosystem, including Ganzirri and Faro brackish lagoons, a close link between the trophic quality of the organic matter pool (suspended matter, particulate organic carbon and nitrogen) and microbially-mediated organic matter turnover was observed, providing insights into the fluxes of carbon, nitrogen and phosphorus potentially mobilized by the microbial community and made available to higher trophic levels.

Among the species playing a relevant role in transitional ecosystems, the noble fan shell, *Pinna nobilis* (L., 1758) is a long-lived species, reaching over 40 years [7]. This habitat-forming species is known to influence significantly both the trophic dynamics and microbial biogeochemistry in coastal environments of the whole Mediterranean [8]. Indeed, as a very large and efficient suspension feeder, this organism filters plentiful volumes of water, removing POM and contributing to benthic-pelagic coupling. This process enhances nutrient cycling and enriches bottom sediments, thus supporting infaunal communities [9]. Additionally, *P. nobilis* shells serve as substrates for diverse epibenthic communities, which in turn are mainly composed of other suspension-feeders, such as sponges [10] and ascidians. Even after death, the empty shells remain anchored in the sediment, continuing to provide habitat for increasing epibiont assemblages which transfer energy and matter from the water column to the sediment [11,12,13]. Moreover, the occurrence of this sensitive species is conventionally considered as a bioindicator of ecosystem health [14].

Unfortunately, a mass mortality event (MME) due to the before-unknown protozoan *Haplosporidium pinnae*, affected in fall 2016 the *P. nobilis* beds throughout the whole Mediterranean Sea [15,16], causing their extinction in marine waters. Introduction in the Mediterranean Sea of this protozoan, native of the northwestern Pacific Ocean, was provided [17]. Mortality was also recorded in association with the presence of *Vibrio mediterranei* [18]. Nevertheless, some populations settled in transitional environments were partially unaffected [18,19,20,21,22]. Among these partially saved areas, Lake Faro has been one of the best investigated sites, starting from the investigation by Giacobbe et al. [23] who first reported the occurrence of *P. nobilis* in this lagoon, demonstrating as fan-shell beds widely occurred in the past, involving a forgotten symbolic role since a pre-Greek age at least. Later, a long-term monitoring of the local population provided quantitative data about its status since 2012 [14] up to the collapse caused by the MME [24], from which only nine adult specimens survived, however representing a potential short-term reservoir for the recovery of this severely threatened species [25].

Preserving, sustaining, and increasing the local population of *P. nobilis* is vital for maintaining the health and stability of these ecosystems, but the relationships linking the occurrence of *P. nobilis* with the water biogeochemistry have never been explored. In this respect, the results of a biogeochemical study, carried out before the MME and never treated before, are reported in this paper, focused on the trophic dynamics and the abundance and metabolic activities of the microbial community in the Faro lagoon waters. The aims of this study were: (i) to assess the trophic dynamics of Lake Faro lagoon and their seasonal variability by analyzing key physicochemical parameters and indicators of water quality, including temperature, salinity, dissolved oxygen, nutrients, chlorophyll-a, and total suspended matter; (ii) to investigate the structure and metabolic potential of microbial communities, focusing on prokaryotic abundance and extracellular enzymatic activities (leucine aminopeptidase, β-glucosidase, and alkaline phosphatase) as functional markers of organic matter degradation and nutrient regeneration; (iii) to examine the occurrence of *P. nobilis*, as a critically endangered bivalve species, in relation to environmental conditions and its potential role in influencing biogeochemical processes and ecosystem functioning, as well as to provide scientific insights to support conservation strategies for *P. nobilis* and promote sustainable management practices for transitional coastal environments.

## 2. Materials and Methods

### 2.1. Study Area and Sampling Stations’ Location

**Figure 1:**
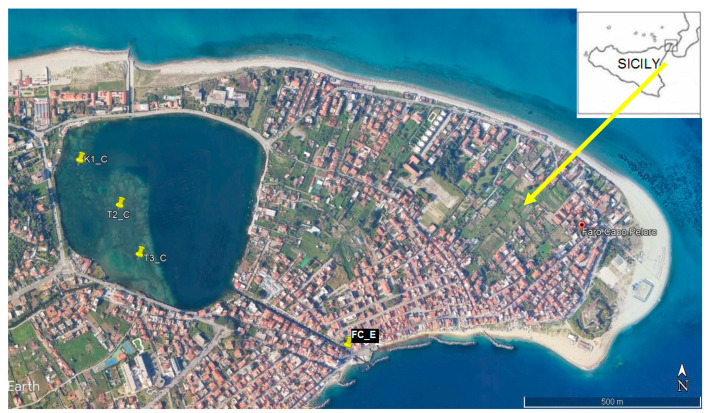
Location of the sampling stations within Faro lagoon (K1_C, T2_C, T3_C) and within Faro canal (FC_E).

Lake Faro (Lat. 38.26861111 N, Long. 15.63694444 E) is a small meromictic coastal basin, belonging to the Cape Peloro lagoon system, between the Tyrrhenian and the Ionian coasts of Sicily (southern Italy) (Figure 1). As a wetland, the whole Capo Peloro lagoon is subject to various protection regimes, starting from its establishment as “Oriented Nature Reserve of Cape Peloro” in 2015. The lagoon was also declared a Site of Community Importance and a Special Protection Zone, according to the European Directives, for its important bio-heritage, being a unique brackish environment as a sanctuary for migratory birds. The lagoon was also designated as a “Heritage of ethnic anthropological interest” because of traditional, not impacting, clam-farming activities [26]. Lake Faro, with a circular shape, covers a 26-ha surface, half of which is characterized by seabed no deeper than 3 m, while the other half has a peculiar funnel shape and reaches a maximum depth of 28 m. Artificial shallow canals connect it with the Straits of Messina (Ionian Sea) and the Tyrrhenian Sea. This morphology is responsible for the peculiar meromictic characteristics, for which this environment has been studied since 1968, because the presence of red waters caused by the development of phototrophic pigmented sulphur bacteria, able to perform photosynthesis even in anaerobic conditions [27,28,29]. The same meromictic regime is responsible for the sharp separation occurring between the highly biodiverse shallow-water platform and the deeper lagoon floors (>7–8 m depth) that are devoid of macroscopic life. Both in the platform and artificial canals, *P. nobilis* beds were recognizable at the time of this investigation, globally counting 480 individuals. Based on the known distribution of the beds, three sampling stations were located in the lagoon (K1_C; T2_C; T3_C) plus one (FC_E), here considered as a control, located in the Faro Canal, connecting this lagoon to the Messina Straits (Figure 1). At these stations, *P. nobilis* specimens were monitored from September 2014 to June 2015, as valuable samples of the whole lagoon and canal populations, as reported in Profeta et al. [30]. Regarding *P. nobilis* census, briefly, SCUBA dives were conducted to identify the areas with the highest concentrations of this species and assess their extent. It was therefore decided to carry out the monitoring along two adjacent linear transects, each 50 m long; in this area too, slugs were placed at the beginning and end of each transect. During the census operations, all live and dead specimens were marked using plastic labels and an identification code based on different colors and the number of holes punched on the surface. In agreement with Siletic and Peharda [31], the labels were fixed to a plastic band and arranged around the base in such a way as not to impede the growth of the specimens but at the same time not to allow them to slip off. For the marking of juvenile specimens, which are smaller and more fragile, plastic pegs not attached to the shell were used, again using an identification code like that described above. The geographical position of the groups of *P. nobilis* was determined by recording the geographical coordinates of a centrally positioned specimen using a portable GPS and noting the arrangement of those surrounding them within a 5 m radius according to the cardinal points.

At the same monitoring stations, seawater surface samples were collected during five sampling periods, performed with a two-month interval, to monitor the temporal evolution of the trophic conditions of the lagoon in relation to the *P. nobilis* specimens’ occurrence.

### 2.2. Environmental Parameters

#### 2.2.1. Physical and Chemical Variables

Temperature (T), salinity (S), dissolved oxygen (DO) and pH values were measured with a multiparametric probe (SeaBird 911 plus, Sea-Bird Scientific, Bellevue, WA, USA). All samples were collected using sterile Niskin bottles and kept at +4 °C until laboratory analysis.

#### 2.2.2. Trophic Variables

The trophic status of the lagoon was assessed through measurements of nutrients, chlorophyll-a and total suspended matter concentrations. Nutrient concentrations (nitrite, NO_2_, nitrate, NO_3_, and orthophosphate, PO_4_) were determined according to Genovese and Magazzù [32]; ammonia (NH_4_) concentrations according to Aminot and Chaussepied [33].

Total and size-fractionated Chl-a concentrations were measured after water filtration on Whatman GF/F filters and extraction in 90% acetone of the recovered material; readings were carried out in a Varian Eclypse spectrofluorometer, previously calibrated with serial standard dilutions of Chl-a from *Anacystis nidulans* (Sigma-Aldrich, St. Louis, MO, USA). The different size fractions (pico-, nano- and micro-sized) were obtained by sequential filtration. The pico-sized fraction (0.2 to 2 µm) includes some of the smallest organisms in the plankton, such as picocyanobacteria and small picoeukaryotes, while the nano- and micro-sized fractions include planktonic organisms belonging to the size categories 2 to 10 µm and 10 to 200 µm, respectively.

Total Suspended Matter (TSM) was evaluated by gravimetric method using a Mettler AT261 electronic microbalance (reliability = 0.01 mg). Particle material was collected by filtering variable volumes of water on pre-combusted (480 °C for 4 h) pre-weighted glass fiber filters (GF⁄F, Whatman, Maidstone, UK), which were further oven-dried at 60 °C for 24 h [5].

The eutrophication index (EI) as an index of the trophic status was calculated from the concentrations of dissolved inorganic nutrients and the Chl-a content recorded at each sampling date [34,35], using the following equation:

EI = 0.279 C_PO4_ + 0.261 C_NO3_ + 0.296 C_NO2_ + 0.275 C_NH4_ + 0.214 C_Chl-a_

Values < 1 suggested low trophic conditions, 1 < x < 3 moderate, and >3 high trophism, respectively.

#### 2.2.3. Microbial Parameters

For total prokaryotic abundance (PA) determinations, samples’ volumes (50 mL) were collected in sterile Falcon tubes and fixed with 0.22 µm prefiltered formaldehyde (2% final concentration) and stored at 4 °C in the dark until laboratory analysis. Variable volumes of sub-samples (0.5 to 5 mL depending on the trophic conditions were filtered on polycarbonate black membranes (25 mm diameter, 0.22 µm pore size) (Nuclepore, Marlborough, MA, USA). After staining with 2, 3-diamidino phenylindole (DAPI [6], at a concentration of 1 mg mL^−1^) and mounting on a microscopic slide, cell counts were performed under a Zeiss Axioplan 2 Imaging epifluorescence microscope (magnification: Plan-Neofluar 100× objective and 10× ocular) equipped with the digital camera AXIOCAM HR (Carl Zeiss AG, Oberkochen, Germany) and with a set of specific filters: G365 excitation filter, FT395 chromatic barrier, and LP420 barrier filter. At least twenty microscopic fields were counted per sample.

Cell abundance (cell mL^−1^) was calculated using the formula:

Cell abundance (cell mL^−1^) = (N × A)/(a × V)

where

N = arithmetic mean of the cell number per microscopic field

A = filtration area (mm^2^)

a = area of the observation field (the whole field or a grid included in the ocular, mm^2^)

V = Volume of the filtered sample (mL)

The metabolic activity of microbial community in the biogeochemical processes was evaluated through potential estimates of extracellular enzymatic activities involved in organic compounds processing (i.e., leucine aminopeptidase; beta-glucosidase and alkaline phosphatase). Microbial enzyme activity rates (leucine aminopeptidase, LAP; beta-glucosidase, GLU and alkaline phosphatase, AP), as markers of the potential ability of the microbial community to decompose peptides and organic phosphates [4], were determined. Ten milliliters of unfiltered sample were incubated, in triplicate, with fixed volumes of l-leucine-4-methylcoumarinylamide hydrochloride (Leu-MCA), methylumbelliferyl (MUF)-beta-glucopyranoside and MUF-phosphate (Sigma) as specific fluorogenic substrates for LAP, GLU and AP, respectively, according to a multi-concentration method. The final concentrations of substrates used in this study ranged from 20 to 200 μmol L^−1^. The fluorescence released by substrate hydrolysis was measured with a F-2000 Hitachi spectrofluorometer (Hitachi, Tokyo, Japan) at time 0, immediately after substrate addition, and 3 h after incubation at “in situ” temperature. Calibration was performed with concentrations from 200 to 800 nmol L^−1^. The compounds 7-amino-4-methylcoumarin or methylumbelliferone were used as the standards for LAP or for GLU and AP, respectively. Data were reported as the maximum velocity of hydrolysis (*V*max) and expressed in terms of nanomoles of Leucine, glucoside or PO_4_ (nmol L^−1^ h^−1^) potentially released per liter and per hour from Leu-MCA or MUF-phosphate, respectively Enzymatic activity rates (in nmol L^−1^ h^−1^) were converted into nanograms of mobilized Carbon, assuming that 1 nmol of substrate hydrolyzed by LAP and GLU activity released 72 ng of C, while 31 ng of P were released from the hydrolysis of 1 nmol of MUF-phosphate.

### 2.3. Statistical Analysis of Data

Analysis of Similarities (ANOSIM) as a non-parametric, permutation-based statistical test, based on a resemblance matrix (in this case, Bray–Curtis similarity). It was applied in order to evaluate differences in the multivariate dataset depending on stations (inner lagoon stations *versus* canal station) and time (among sampling periods) and the relative importance of space *versus* time in shaping environmental and microbial patterns.

In addition, a one-way similarity percentage procedure (SIMPER routine) was used to assess the percentage contribution of each variable to the Bray–Curtis similarity between the groups of samples. Multivariate Principal Component Analysis (PCA) was computed; it is a multivariate ordination technique that reduces a complex dataset of variables into a smaller number of orthogonal components, each representing major gradients of variability. Unlike ANOSIM, PCA allows the identification of a set of principal components, which represent the directions of maximum variance in the data, and of the relationships among variables and their joint response to environmental forcing.

Bi-dimensional representations of the statistical comparisons among stations and sampling times were obtained by non-parametric multidimensional scaling (nMDS) performed on Bray–Curtis similarity matrices (four-root transformed data). All statistical tests were carried out using the PRIMER v.6 software package [36] (Primer-E). In addition, Pearson’s correlation coefficients among the environmental and microbial parameters were calculated using a standard Microsoft Excel software statistical function.

## 3. Results

### 3.1. Environmental Physical and Chemical Variables

**Figure 2:**
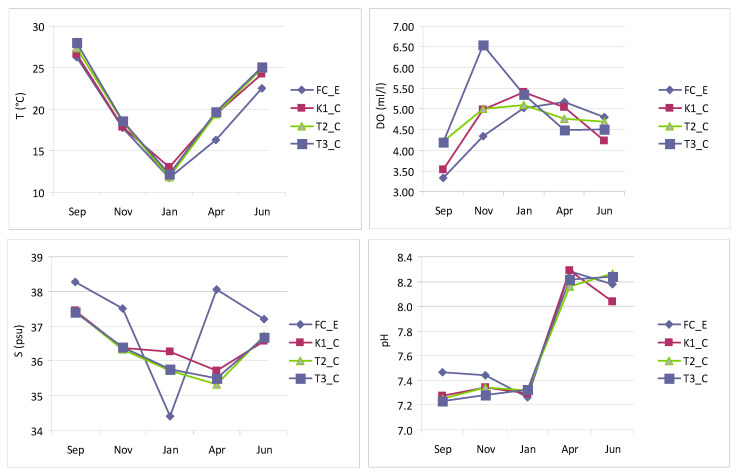
Physical-chemical parameters measured in Faro lagoon (stations K1_C, T2_C, T3_C) and control station (FC_E): temperature (T), salinity (S), dissolved oxygen (DO) and pH.

During the monitored period, T values in the lagoon ranged from 12 °C (T2_C, in January) to 28 °C (T3_C, in September), showing the same trend at all three stations (Figure 2). At the control station (FC_E), the temperature was more variable, starting from 26 °C in September, reaching a minimum of 12 °C in January, and maintaining slightly lower values than in the lagoon up to the last record, in June.

Salinity varied in the lagoon between 35 in April (T2_C) and 37 in September (K1_C) showing almost the same trend at all stations, although slightly higher values were found in January and April at the more inner station (K1_C) than elsewhere. The control station showed a very different trend, with very more marked fluctuations, ranging from 38 in September to 34 in January.

As far as DO concentration is concerned, a similar trend characterized the lagoon stations K1_C and T2_C together with the control, while the station T2_C showed a different trend. At this latter, in fact, in November was observed a peak of 6.54 mL/L, much higher than the maximum recorded at the other stations, in April (5.34 mL/L in K1_C). The minimum value, 3.53 mL/L was found in September (St. K1_C).

As regards pH, both lagoon stations and the control showed a similar trend, with low values in September-January (minimum 7.23 in T3_C) followed by a sharp increase in April–June (maximum 8.29 in FC_E).

### 3.2. Trophic Conditions

**Figure 3:**
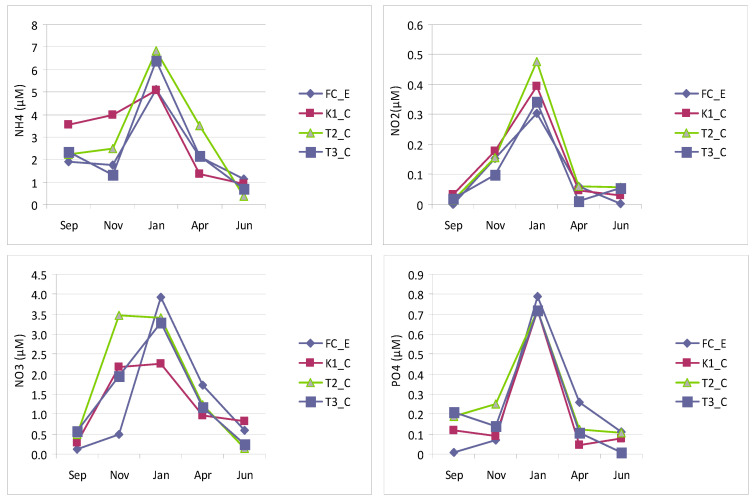
Nutrient concentrations measured in Faro lagoon (stations K1_C, T2_C, T3_C) and canal station (FC_E): ammonia (NH_4_), nitrites (NO_2_), nitrates (NO_3_) and orthophosphate (PO_4_).

Nutrient concentrations (Figure 3) were characterized by the predominance of NH_4_, whose maximum (6.83 µM in January) and minimum (0.38 µM in June) values were both recorded at station T2_C. All stations exhibited a similar temporal pattern, except for K1_C, where NH_4,_ concentrations in September and November were higher than at the other sites. NO_2_ concentrations showed a largely consistent trend across all stations, ranging from 0.00064 µM (station FC_E, September) to 0.48 µM (T2_C, January). In contrast, NO_3_ concentrations exhibited a different pattern: in September, concentrations were low and similar among the stations (0.11–0.57 µM), then increased in November at K1_C and T2_C, reaching maxima of 2.17 µM and 3.47 µM, respectively. These values remained high in January, before declining to minima in June. At stations T3_T and FC_E, the highest NO_3_ values were observed in January (3.28 µM and to 3.92 µM, respectively), followed by near-zero concentrations at station T3_C in June.

PO_4_ concentrations displayed similar seasonal variations, ranging from 0.01 µM to 0.79 µM, both recorded at the control station (FC_E), in September and January, respectively (Figure 3).

**Figure 4:**
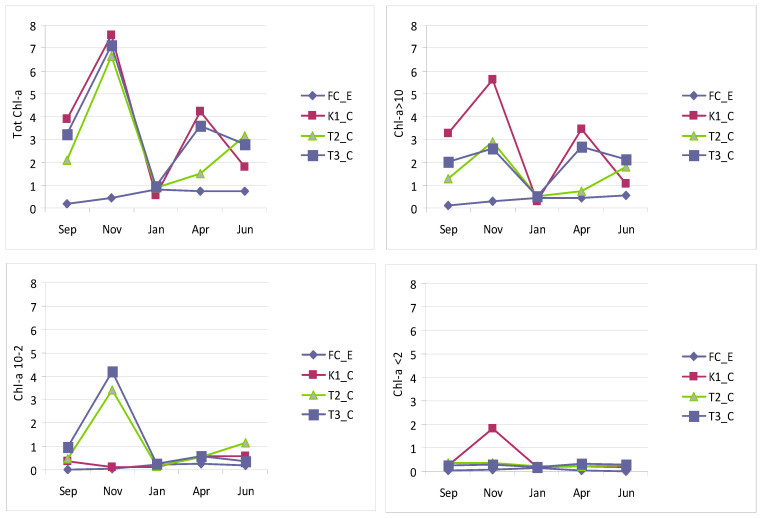
Total and size-fractionated chlorophyll-a measured at the Faro lagoon (stations K1_C, T2_C, T3_C) and control station (FC_E).

Chl-a concentrations in the lagoon waters showed wide fluctuations, ranging from 0.55 µg L^−1^ in January to 7.54 µg L^−1^ in November (both recorded at station K1_C). These values fall within a range that classifies Lake Faro lagoon as a moderately trophic water body (Figure 4). Stations K1_C and T3_C exhibited similar temporal patterns, with a main peak in November and a secondary one in April, corresponding to the autumn and spring blooms, respectively. Conversely, at station T2_C, the spring bloom was absent, and only a secondary peak was recorded in January. At the control station FC_E, Chl-a concentrations remained below 1 µg L^−1^, reaching minimum values in September.

Size-fractionated Chl-a analysis revealed the predominance of the micro-sized (>10 µm) fraction, which at station K1_C showed the wider range, i.e., from 0.30 µg L^−1^ (January) to 5.61 µg L^−1^ (November). The nano-sized (10–2 µm) fraction ranged between 0.09 µg L^−1^ and 4.199 µg L^−1^ (both in November, at stations K1_C and T3_C, respectively). The pico-sized (<2 µm) fraction was negligible, ranging from 0.16 µg L^−1^ (January) to 1.82 µg L^−1^ (November), both recorded at station K1_C. Moreover, whereas the micro-sized fraction followed the same temporal pattern as the total Chl-a, both the pico- and nano-sized fractions exhibited a single autumn peak, at stations T3_C and T2_C and at station K1_C, respectively.

**Figure 5:**
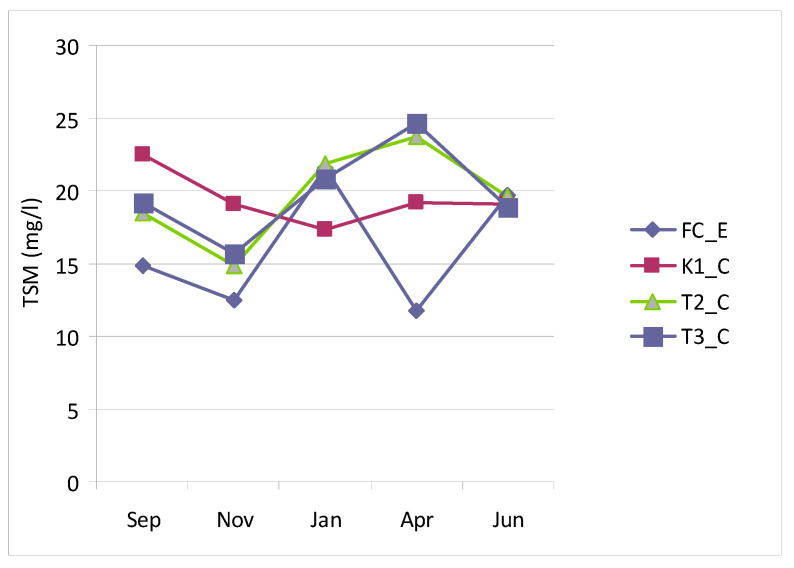
Total Suspended Matter concentrations recorded in the Faro lagoon (stations K1_C, T2_C, T3_C) and control station (FC_E).

In Lake Faro, TSM concentrations ranged from 14.84 mg L^−1^ (T2_C November) to 24.60 mg L^−1^ (T3_C in April) (Figure 5). Stations T2_C and T3_C displayed nearly identical trends, whereas station K1_C showed higher TSM concentration during September–November, which decreased over time (January–June), contrasting with the increasing trend observed at stations T2_C and T3_C. In the canal (station FC_E), TSM concentrations were generally lower (11.79–21.55 mg L^−1^) and exhibited more pronounced fluctuations. The control station mirrored the lake’s trends during the early period (September–January) but diverged later (April–June), when TSM reached a minimum at FC_E and a maximum within the lake.

**Figure 6:**
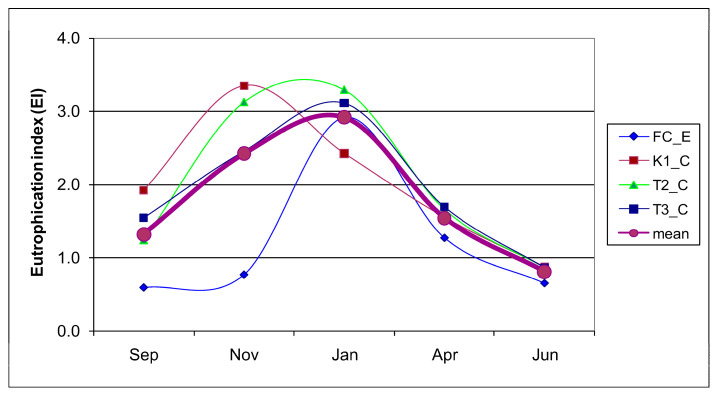
Eutrophication index calculated at each Faro station (lagoon stations K1_C, T2_C, T3_C) and control station FC_E) per each sampling and as the mean value from the lagoon stations.

The mean EI calculated for the lake stations (Figure 6) indicated low trophic conditions in January (EI < 1), moderate trophism in September and April (EI = 1–3), and borderline high values in November–January, when this index reached 3.0, indicating a high trophic level. These high values reflected asynchronous peaks of trophic parameters at the three stations, occurring in November at station K1_C and in January at stations T2_C and T3_C. At the control station (FC_E), EI values were consistently lower, particularly in September and November, both indicating low trophic conditions.

### 3.3. Prokaryotic Abundance and Metabolic Activity

**Figure 7:**
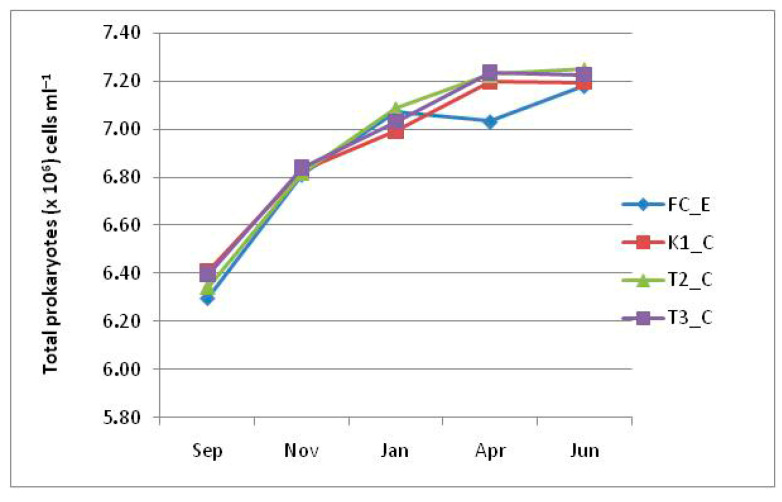
Total prokaryotic abundance recorded in the Faro lagoon (stations K1_C, T2_C, T3_C) and control station (FC_E).

Total PA showed similar trends at all lagoon stations, globally ranging from a minimum of 10^6^ cells mL^−1^ in September to a maximum of 10^7^ cells mL^−1^ in June. The same behavior was also observed at the control station, except for a moderate and temporary decrease in April (Figure 7).

**Figure 8:**
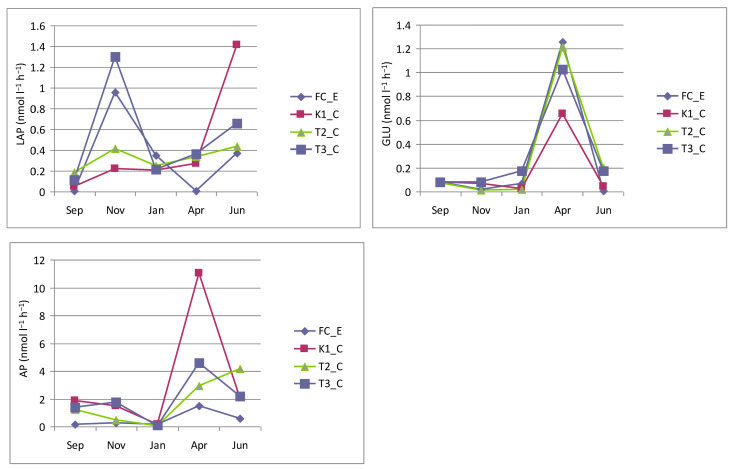
Enzyme activity rates (LAP, GLU and AP) measured in the Faro lagoon (stations K1_C, T2_C, T3_C) and Faro Canal station (FC_E).

Enzymatic activity assays (Figure 8) showed that AP was consistently the dominant enzyme, with activity rates in the lake ranging from 0.05 nmol L^−1^ h^−1^ (station T2_C, January) to 11.03 nmol L^−1^ h^−1^ (station K1_C, April). In the canal, AP activity was generally lower, except in January, when values were comparable to the lake stations; in most cases, activity approached zero. A similar overall trend was observed at all sites, with a peak in April that was most pronounced at station K1_C, less marked at station T3_C, and faint at station FC_E. At station T2_C, however, the maximum occurred later, in June.

As far as GLU is concerned, both lagoon and control stations showed very low values between September and January (minimum 0.008 nmol L^−1^ h^−1^ at station FC_E, January), followed by a temporary and sharp peak in April, when maximum values (1.256 nmol L^−1^ h^−1^) were recorded at station FC_E.

Compared to AP and GLU, LAP activity exhibited more irregular fluctuations, with peaks in November and June at all stations, including the control. Minimum LAP activity was observed in September (0.006 nmol L^−1^ h^−1^) and April (0.01 nmol L^−1^ h^−1^) at station FC_E, whereas the maximum was recorded in June at station K1_C.

### 3.4. Statistical Elaboration of the Dataset

The ANOSIM performed on the complete dataset revealed significant differences both among stations (Rho = 0.691, *p* = 0.2%) and among months (Rho = 0.713, *p* = 0.1%), whereas the month × station interaction term was not significant (Rho = 0.422, *p* = 1.6%) (Supplementary Table S1). This indicates that seasonality exerted a stronger and more consistent influence on microbial and trophic parameters than small-scale spatial heterogeneity; nonetheless, spatial gradients related to the lagoon confinement and water exchange were still detectable.

The SIMPER analysis indicated that, on a spatial scale, stations T2_C and T3_C were highly similar to each other, with a squared distance (D^2^) of 4.67, whereas stations FC_E and K1_C were markedly different (D^2^ = 27.23) (Table S1). At stations FC_E and K1_C, the variables contributing most to the observed differences were primarily related to autotrophic processes, whereas at stations T2_C and T3_C heterotrophic processes (particularly LAP activity) accounted for the dissimilarities. At station K1_C, both autotrophic and heterotrophic variables contributed substantially to the observed dynamics.

On a temporal scale, the SIMPER analysis highlighted close similarities between the April and June samplings (D^2^ = 19.80), as well as between September and June (D^2^ = 21.03). In June, heterotrophic variables, including PA and LAP (April *vs.* June) and GLU and AP (September *vs.* June) contributed most to these differences (see Table S1). Similarly, heterotrophic processes were responsible for the differences observed between April and September (PA, GLU) and between April and January (AP, GLU, NO_2_, PO_4_). In the remaining comparisons, autotrophic variables (total and 10–2 µm size-fractionated Chl-a) accounted primarily for the observed dissimilarities.

**Figure 9:**
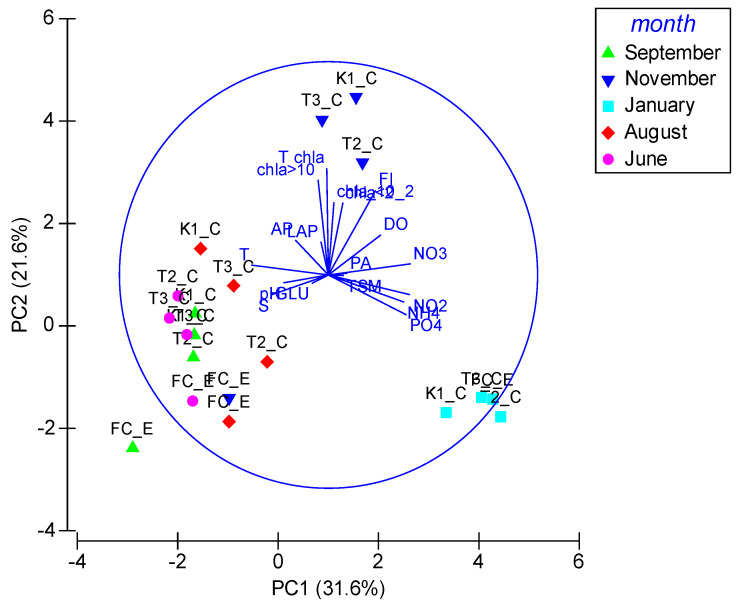
Principal Correlation Analysis computed on the whole dataset using months and stations (K1_C; T2_C, T3_C; FC_E) as the main variables.

Multivariate PCA highlighted that PC1 and PC2 jointly explained a substantial fraction of the total variance, as much as 53.2% (Figure 9). It indicated a close association between AP and LAP, whose displayed opposite trends with respect to PA and GLU. GLU was associated with S and pH. PA was negatively affected by T, while TSM was associated with the nutrients pool. T was directly correlated with Chl-a content, but negatively with PA. Globally, significant associations between specific enzymatic activities with environmental gradients such as T, S, pH, and nutrient availability were observed; an opposite behavior of microbial metabolic indicators *versus* primary production proxies (chl-a fractions) along seasonal gradients was found, suggesting a separation between autotrophic and heterotrophic processes.

**Figure 10:**
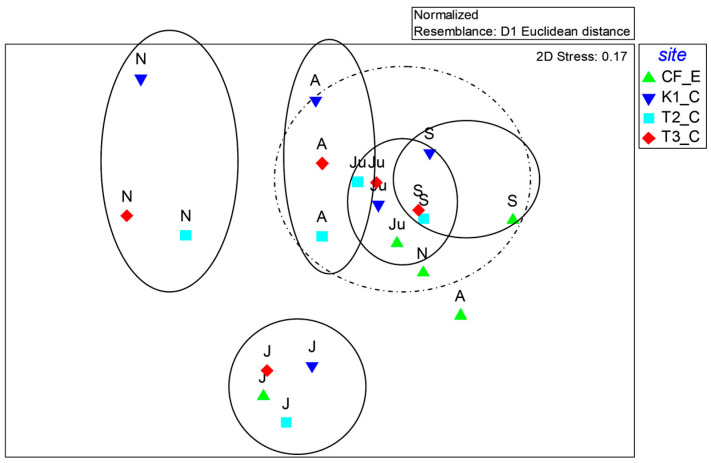
Outputs of the n-MultiDimensional Scaling Analysis overlapped with Cluster Analysis performed on the whole dataset. S = September; N = November; J = January; A = April; Ju = June. The dashed line includs three sub-clusters.

Non-parametric MultiDimensional Scaling analysis (nMDS), overlapped with Cluster analysis, highlighted a clear seasonal pattern in environmental and microbial parameters. Warm-period samplings (April, June, and September) clustered closely together, in contrast to those in November and January, which remained distinct (Figure 10). Within each sampling period, stations T2_C and T3_C generally clustered together, while station FC_E remained relatively isolated.

### 3.5. Occurrence of P. nobilis in Relation to Microbial Parameters

Based on the contextual investigation reported in Profeta et al. [30], the *P. nobilis* population in Lake Faro exhibited a random distribution pattern, with individuals mostly occurring as isolated specimens and, more rarely, aggregated in small patches. Among the four investigated stations, most individuals were recorded at stations FC_E and T2_C, with the highest abundances in September and November and lower values in April and June. Dead individuals were also observed, particularly at stations K1_C and T3_C (Table 1).

The density of *P. nobilis* was generally low, ranging from 0.25 to 6 individuals per 100 m^2^, particularly at station T3_C, where a single specimen recorded in September was found dead in April. The highest abundance was observed in the Faro canal (station FC_E), with densities ranging between 4 and 45 individuals per 100 m^2^. No significant temporal trend was detected (*p* > 0.5).

**Figure 11:**
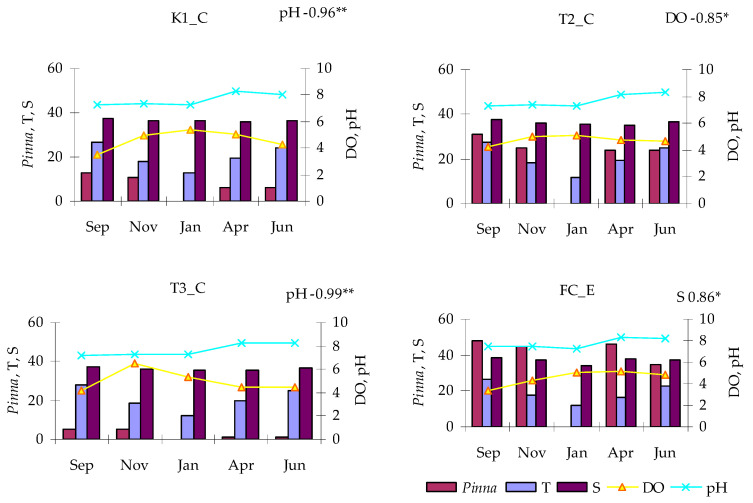
Abundance of *Pinna nobilis* in relation with environmental parameters. Significant correlations are shown (* *p* < 0.05; ** *p* < 0.01).

**Figure 12:**
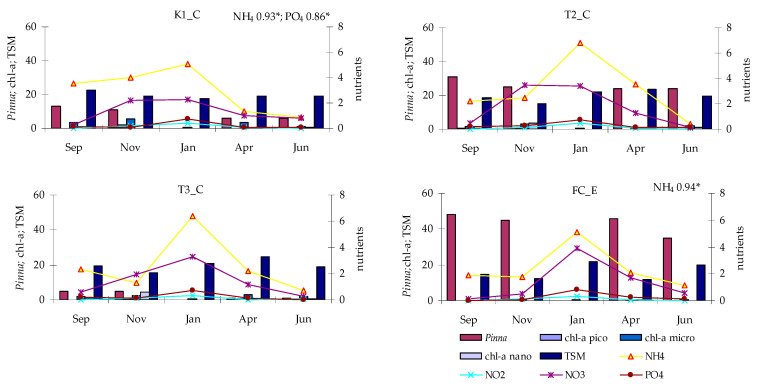
Abundance of *Pinna nobilis* in relation to trophic parameters. Significant correlations are shown (* *p* < 0.05).

Potential relationships linking the occurrence of *P. nobilis* with microbial parameters were assessed by calculation of Pearson’s correlation coefficients at each station (Supplementary Table S2). *P. nobilis* density correlated positively with S at station FC_E, changing with the sea, negatively with pH at stations K1_C and T3_C and negatively with DO at station T2_C (Figure 11). Positive correlations were found between *P. nobilis* and nutrient concentrations (e.g., with PO_4_ and NH_4_ at station K1_C, and with NH_4_ at station FC_E) (Figure 12).

**Figure 13:**
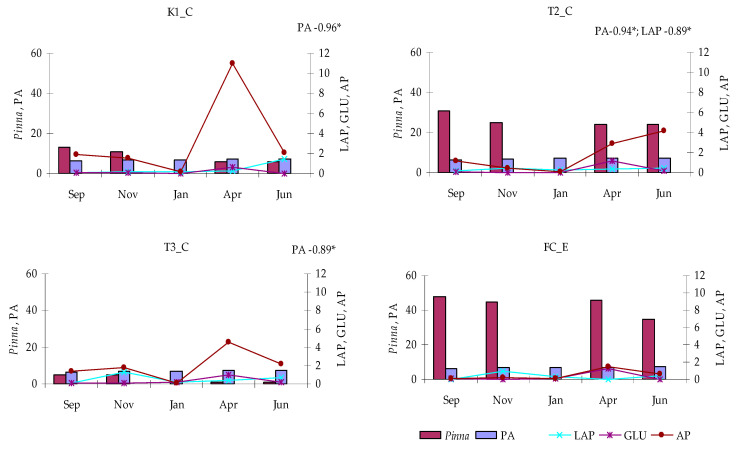
Abundance of *Pinna nobilis* in relation to microbial parameters. Significant correlations are shown (* *p* < 0.05).

Conversely, negative relationships were detected between *P. nobilis* and PA at all lagoon stations; with LAP at station T2_C (Figure 13). These results suggest that the distribution of *P. nobilis* was not directly related to microbial heterotrophic activity.

## 4. Discussion

### 4.1. Environmental Context and Microbial Dynamics

Transitional coastal environments, acting as ecotonal systems between terrestrial and marine realms, are characterized by dynamic gradients in salinity, temperature, and nutrient availability [1]. These features make them biodiversity hotspots but also highly sensitive to anthropogenic pressures such as urbanization and seasonal tourism. In this framework, the occurrence of the giant bivalve *P. nobilis* is of particular relevance due to its sensitivity to habitat quality and its key role in benthic–pelagic coupling [8,14]. Following the mass mortality event (MME) that brought the species to the threat of extinction—leaving remnant populations in some transitional environments—the role of brackish basins like Lake Faro in the Peloro Lagoon as “last sanctuaries” has become crucial, emphasizing the need for detailed knowledge of their environmental characteristics.

The present study, aimed at evaluating microbial biogeochemistry and trophic dynamics during the pre-MME of *P. nobilis* in Peloro Lagoon and potential relationships between environmental characteristics and the abundance of this bivalve, revealed overall good environmental conditions based on physicochemical parameters.

Seasonal variability was evident, with warmer months (April–June) characterized by higher T and reduced DO concentrations. Some difference emerged between the lake stations and the control, together with a faint gradient separating the innermost station (K1_C) from the other two more central stations (T2_C, T3_C). Unexpectedly, the control station (FC_E), despite its lesser degree of confinement, showed more marked temporal variations than the lake stations. Among inorganic nitrogen pools, ammonia ions were predominant, suggesting strong regeneration processes, which is consistent with the nature of shallow lagoon of the examined environment [37]. The observed uncoupling between peaks in nutrient concentrations (in winter) and chl-a (in November and April) indicated that phytoplankton biomass is controlled not exclusively by nutrient availability, but that additional factors including light, grazing, or hydrodynamics may act as drivers of its variability. In general, the observed conditions coincided with enhanced microbial heterotrophic activity, suggesting a shift in ecosystem functioning. EI values indicated moderate trophic conditions overall, with peaks in January and November, particularly at the innermost stations (T2_C and K1_C).

Microbial abundance, ranging from 10^6^ to 10^7^ cells mL^−1^, was higher during warmer months and at the inner lake stations. Enzymatic activity rates were dominated by alkaline phosphatase (AP), followed by leucine aminopeptidase (LAP), whereas β-glucosidase (GLU) showed lower activity. No peaks of AP in correspondence of minimum PO_4_ concentrations were observed, suggesting that the expression of AP activity was not strictly stimulated by phosphorus limitation but that this enzyme could have a dual role, providing also a carbon source [38]. In the present investigation, enzyme activity estimates were not reported in terms of cell-specific activity, i.e., by normalizing bulk enzyme rates to PA; actually, normalization to cell abundance could bias data interpretation because enzymatic synthesis is affected, other than by environmental conditions, by the physiological cell status and can vary strongly among microbial taxa [39]. It is also true that many extracellular enzymes remain active after being released into the environment, either dissolved in the water column or adsorbed to particles; because these enzymes can persist long after active secretion or cell lysis, enzyme activity may therefore suggest a previous microbial production. Particle association may also protect enzymes from degradation, prolonging their lifetime and allowing sustained activity even when a low living microbial biomass is present [40]. Uncoupled trends eventually observed between measured enzyme activities and PA therefore underline the importance of considering both living microbial communities and the environmental enzyme pool when interpreting biogeochemical rates. Anyway, the observed enzymatic pattern indicated preferential degradation of organic phosphates and proteins, consistent with findings from other Sicilian lakes [4,6]. Overall, our results highlighted the strong influence of seasonal dynamics on microbial and trophic processes in transitional basins. Similar findings were reported by Pala et al. [41] in a Mediterranean lagoon, Sacca di Goro; there, trophic conditions and abiotic factors such as temperature, ammonium, pH were found to significantly influence bacterial distribution, confirming that lagoon ecosystems exhibited strong spatial and seasonal gradients affecting microbial community patterns. In turn, differences in the microbial loop functioning depending on organic matter refractory or labile biochemical composition—such as those observed by comparing coastal lagoons dominated by a classical “detritus” chain with those acting as a “source” system—were reported to result in a different ability of channeling carbon to higher trophic levels [42]. Although highly productive Mediterranean coastal ecosystems including lagoons are dominated by macrofauna and macrophytes, the interaction between microbial and microbial organisms—supporting quick nutrient cycling and preventing organic matter accumulation—has been suggested to be relevant for accurate conservation and management of these vulnerable environments [43]. Furthermore, the responsiveness of microbial parameters to seasonal nutrient fluxes supports their use as functional indicators of ecosystem health, as also observed in other aquatic systems [44].

### 4.2. Influence of P. nobilis on Water Biogeochemistry and Ecosystem Functioning, Links Between Environmental Microbiological Conditions and P. nobilis Health

It must be noted that this research is the first study trying to associate prokaryotic dynamics with the occurrence of *P. nobilis* in Peloro Lagoon. Indeed, a better understanding of the interplay between microbial communities, *P. nobilis* ecology, and environmental gradients is essential to support effective conservation and management strategies for this endangered species. Some limitations in our experimental design must be acknowledged: first, sampling was restricted to surface waters, limiting the interpretation of biogeochemical processes and benthic pelagic coupling occurring at depths and near the sediment–water interface in this permanently stratified lagoon; second, the temporal replication was limited to five samplings only, weakening the interpretation of ecosystem functioning and of the interactions between microbial parameters and *P. nobilis;* third, the performed enzyme activity measurements gave potential activity rates referred to high substrate concentrations that might differ from those present in nature. According to these constraints that could potentially affect data interpretation, the effects played by *P. nobilis* on microbial and trophic dynamics shown by the recorded dataset must be framed as hypotheses rather than actual mechanisms explaining ecosystem functioning; as a consequence, no direct cause–effect relationship can be formulated and caution must be taken in interpreting data regarding this feature. Nevertheless, weak or negative relationships observed between *P. nobilis* density and microbial enzymatic activities in surface waters led us to suppose a limited support of *P. nobilis* excreta to the heterotrophic microorganisms and consequent organic matter decomposition processes in the water column, rather reflecting a prevalent interaction of *P. nobilis* with the water–sediment interface.

As detailed in Profeta et al. [30], *P. nobilis* distribution was spatially heterogeneous, with higher densities in the Faro Canal and lower densities at the inner lake stations. Specimen size varied accordingly, averaging 110 mm in the canal and 153 mm in the lagoon, suggesting that the canal played a preeminent role as a recruitment site, whereas the lagoon provided more favorable conditions for growth and survival.

Bearing in mind the potential limitation of speculative assumptions of links between microbial communities and the presence of this bivalve species, negative relationships between the abundance of *P. nobilis* and enzymatic activity rates might suggest that areas with higher densities of the pen shell tend to exhibit lower microbial enzymatic activity in the water column. This could be explained by the fact that, by removing suspended particles and promoting benthic–pelagic coupling, *P. nobilis* may reduce organic matter flux in the water column, limiting microbial demand for enzymes involved in protein and carbohydrate degradation (e.g., LAP and GLU). Moreover, all pen shell species are suspension-feeders that through their filtering activity may reduce the availability of organic substrates for heterotrophic microbes, leading to lower enzymatic activity. Conversely, this bivalve primarily interacts with particulate organic matter and nutrients at the water–sediment interface rather than stimulating microbial activity in the water column. Therefore, the presence of *P. nobilis* could favor clearer waters, reducing organic load and consequently microbial heterotrophic metabolism and trophic stress. This aligns with its role as a bioindicator of good environmental status. Briefly, negative correlations might imply that *P. nobilis* does not enhance microbial enzymatic activity in surface waters but may instead contribute to ecosystem stability by reducing organic load and promoting nutrient cycling at the sediment level. Positive correlations with nutrient concentrations (e.g., PO_4_ and NH_4_), indeed, seemed to suggest a potential role of *P. nobilis* in the mobilization of nutrients, consistent with previous studies emphasizing its contribution in benthic–pelagic coupling and nutrient cycling [45,46]. 

Multivariate analyses (ANOSIM, SIMPER, PCA, nMDS) revealed that seasonal variability exerted a stronger influence on microbial and environmental parameters than spatial differences. ANOSIM findings directly addressed two objectives of the study, namely assessing trophic dynamics and their seasonal variability, and investigating microbial abundance and metabolic potential in relation to environmental conditions. In addition, SIMPER analysis showed that heterotrophic processes dominated during warmer periods, conversely to autotrophic variables (e.g., Chl-a) that prevailed in cooler months. PCA indicated clear associations between enzymatic activities and environmental gradients related to T and S; this analysis helped to identify the main drivers regulating microbial biogeochemical functioning in the lagoon and to clarify how nutrient pools, suspended matter and phytoplankton biomass were linked to microbial metabolism. Microbial processes in Faro lagoon contributed significantly to nutrient cycling, organic matter degradation, and potential carbon sequestration. Moreover, even in the absence of strong direct correlations, PCA helped to contextualize the occurrence of *P. nobilis* within broader environmental and biogeochemical gradients.

Globally, these results underscored the importance of integrating microbial and trophic indicators when assessing ecosystem functionality and resilience. The meromictic nature and seasonal stratification of this lagoon promoted the formation of distinct microbial niches, with implications for greenhouse gas emissions and pollutant breakdown [47]. Anthropogenic influences, including seawater inflow and shellfish farming, further modulated these dynamics. Since brackish lagoons serve as refuges for *P. nobilis* larval spawning and recruitment [48], the collapse of these populations underscores the urgency of species conservation. Another important aspect that should be considered addressing *P. nobilis* population is the modulation of its abundance played by predators. Adult populations, as that here studied, are unaffected by predation, only juveniles being vulnerable because of their thin shell (e.g., [49,50]). Evidence of this has also been reported in a previous study on the survival rate of the same Faro lagoon population [14]. Moreover, although predation on adults has been suggested as possible on individuals weakened by *Aplosporidium pinnae* during the recent mass mortality event [20], no evidence of predation on dying specimens has been reported from the monitored Faro lagoon population [51]. 

Links between the microbial composition of environmental microbiota and pathogens and the presence and health status of *P. nobilis* were previously explored in a few studies. Pavlinec et al. [52] analyzed the bacterial community hosted in the *P. nobilis* tissues from the Eastern Adriatic coast, using 16S rRNA sequencing, showing the predominance of the genera *Aestuariibacter*, *Aliivibrio*, *Alteromonas*, *Pseudoalteromonas*, *Marinobacter*, *Rubritalea*, *Thalassospira* and members of the *Vibrio splendidus* clade. Spatial differences in the bacterial composition were correlated to water temperature fluctuations. This study suggested how microbial composition varied in relation to the habitat and the environmental temperature, affecting the physiological condition of *P. nobilis*. Lattos et al. [46] in the digestive tract of diseased individuals examined using a 16S rRNA sequencing and NGS approach detected the presence of *Vibrio* spp., *Aliivibrio* spp., *Photobacterium* spp., *Pseudoalteromonas* spp., *Psychrilyobacter* spp. and *Mycoplasma* spp., linking the presence of opportunistic pathogens like *Vibrio* spp. to the occurrence of mass mortality events. Lattos et al. [53] highlighted the involvement of bacteria belonging to the *Vibrio* genus in the mass mortality of *P. nobilis*, with isolation of the species *V. mediterranei* and *V. splendidus* in moribund specimens. Grau et al. [54] integrated the study of the habitat and environmental conditions with the occurrence of mortality events in *P. nobilis* due to *Haplosporidium pinnae*, *Mycobacterium* spp. and *Vibrio* spp. Overall, these studies indicated that there were significant relationships between the structure of the microbial communities hosted by *P. nobilis* and in the surrounding environment, the presence of opportunistic pathogens (i.e., *Vibrio*) and the environmental conditions such as temperature, salinity and habitat properties (i.e., seagrasses, bottom characteristics).

## 5. Conclusions

By integrating trophic and microbial data with the occurrence of *P. nobilis*, this research contributes to a deeper understanding of ecosystem functioning and offers a framework for preserving biodiversity in vulnerable coastal habitats. This study confirms the active role of microbial communities in regulating biogeochemical processes in Faro lagoon and highlights the limited yet detectable influence of *P. nobilis* on surface microbial dynamics. Strong seasonal patterns suggest that climate variability and temperature fluctuations substantially affect microbial metabolism and overall ecosystem functioning. Overall, the combined application of multivariate analyses demonstrates that seasonality is the main driver of microbial and trophic dynamics in Lake Faro lagoon, modulating nutrient availability, microbial metabolism, and phytoplankton biomass. These complimentary statistical analyses strengthen the integrated interpretation of trophic, microbial, and biological data by showing that microbial and trophic dynamics are primarily driven by seasonal variability, with secondary spatial effects linked to lagoon confinement; and that *P. nobilis* distribution develops within an environmental setting primarily shaped by seasonal variability rather than by short-scale spatial differences, supporting the hypothesis that lagoon-scale ecosystem functioning and stability are critical for the persistence of this endangered species. Therefore, microbial and trophic dynamics create a biogeochemical context within which *P. nobilis* populations persist, confirming the view of transitional lagoons as potential refuges for *P. nobilis* and confirming the value of microbial functional markers as an effective tool for assessing ecosystem health and supporting conservation strategies in Mediterranean transitional environments.

Further research should focus on vertical profiling of microbial communities, more detailed analysis of benthic–pelagic interactions, and the functional genomics of microbial taxa associated with *P. nobilis*. To implement research on the habitat–organism relationships, different features could be investigated: (i) a comparative analysis should be carried out between the environmental microbiota and the microbiota associated with *P. nobilis* tissues or organs; (ii) the application of shotgun analytical approaches might contribute to increase knowledge of the microbial composition besides 16S rRNA sequencing; (iii) the number of the environmental parameters examined could be expanded, including the presence of heavy metals/additional microbial indicators. Nevertheless, insights from the present study improve our understanding of Faro lagoon and support the sustainable management and conservation of this unique habitat.

## Figures and Tables

**Table 1:** Distribution of *Pinna nobilis* in Lake Faro. The number of Live (L) and Dead (D) individuals per 100 m^2^ is reported.

	Sep		Nov		Apr		Jun	
	L	D	L	D	L	D	L	D
FC_E	45	3	43	2	40	6	31	4
K1_C	5	8	5	6	6	0	6	0
T2_C	30	1	22	3	24	0	24	0
T3_C	1	4	1	4	0	1	0	1

## Data Availability

The original contributions presented in this study are included in the article/Supplementary Materials. Further inquiries can be directed to the corresponding author.

## Supplementary Materials

The following supporting information can be downloaded at: https://www.mdpi.com/article/10.3390/microorganisms14020423/s1, Table S1: Outputs of Similarity Percentage (SIMPER) analysis computed on a seasonal and spatial scale using the PRIMER 6.0 software (S = September, N= November; J = January; A = April; Ju = June). Table S2: Pearsons’ correlation coefficients computed per each station among biological and environmental parameters (* *p* < 0.05; ** *p* < 0.01).
