# Supplementary material for: Trophic and Microbial Dynamics in a Mediterranean Transitional Ecosystem (Lake Faro, Southern Italy): Implications for Pinna nobilis Conservation
Source: Microorganisms. 2026 Feb 11;14(2):423. doi: 10.3390/microorganisms14020423 (PMC12942930; doi:10.3390/microorganisms14020423)
Supplement: Supplementary file 1 [file microorganisms-14-00423-s001.zip › Supplementary Table S1.pdf]

microorganisms

MDPI

*Article*

# Trophic and Microbial Dynamics in a Mediterranean Transitional Ecosystem (Lake Faro, Southern Italy): Implications for *Pinna nobilis* Conservation

**Gabriella Caruso [1,*], Salvatore Giacobbe [2], Filippo Azzaro [1], Franco Decembrini [1], Marcella Leonardi [1], Giovanna Maimone [1], Adriana Profeta [2] and Paola Rinelli [2]**

**Supplementary Table S1.** Outputs of SIMPER analysis (S=September, N= November; J=January; A=April; Ju=June)

**SIMPER on a seasonal scale**

| | Average squared distance | Variable | Av.Sq. Dist | Sq.Dist/SD | Contrib% | Cum.% | Trophic pathway |
|---|---|---|---|---|---|---|---|
| Groups S & N | 35.15 | DO | 4.77 | 1.04 | 13.56 | 13.56 | auto+hetero |
| | | Fl | 4.60 | 1.43 | 13.09 | 26.65 | |
| | | chla_20_5 | 4.43 | 0.50 | 12.59 | 39.24 | |
| | | chla_10_2 | 3.96 | 0.86 | 11.26 | 50.50 | |
| | | LAP | 3.88 | 0.88 | 11.03 | 61.53 | |
| Groups S & J | 49.22 | T | 7.69 | 7.64 | 15.62 | 15.62 | hetero |
| | | $NO_2$ | 6.63 | 2.44 | 13.47 | 29.09 | |
| | | S | 5.72 | 0.87 | 11.61 | 40.70 | |
| Groups S & A | 32.35 | PA | 6.33 | 6.26 | 19.55 | 19.55 | hetero |
| | | GLU | 5.73 | 2.02 | 17.72 | 37.27 | |
| | | pH | 4.25 | 5.46 | 13.13 | 50.40 | |
| | | AP | 3.90 | 0.63 | 12.05 | 62.45 | |
| Groups S & Ju | 21.03 | PA | 6.95 | 7.95 | 33.04 | 33.04 | hetero |
| | | LAP | 3.82 | 0.69 | 18.18 | 51.22 | |
| | | pH | 3.80 | 2.85 | 18.09 | 69.31 | |
| Groups N & J | 49.63 | T Chla | 5.72 | 1.43 | 11.53 | 11.53 | auto |
| | | chla_10_2 | 5.46 | 0.84 | 11.01 | 22.54 | |
| Groups N & A | 44.55 | GLU | 6.15 | 1.90 | 13.80 | 13.80 | auto+hetero |
| | | chla_20_5 | 4.67 | 0.51 | 10.49 | 24.29 | |
| Groups N & Ju | 38.85 | Fl | 4.99 | 1.39 | 12.85 | 12.85 | auto |
| | | chla_20_5 | 4.78 | 0.50 | 12.29 | 25.14 | |
| | | chla_10_2 | 4.14 | 0.73 | 10.65 | 35.79 | |
| Groups J & A | 45.20 | AP | 5.87 | 0.68 | 12.98 | 12.98 | hetero |
| | | GLU | 5.81 | 1.92 | 12.85 | 25.83 | |

| | | | | | | | |
|---|---|---|---|---|---|---|---|
| | | $NO_2$ | 5.64 | 2.47 | 12.48 | 38.31 | |
| | | $PO_4$ | 5.33 | 5.11 | 11.80 | 50.11 | |
| Groups J & Ju | 44.55 | $NH_4$ | 7.68 | 2.19 | 17.25 | 17.25 | hetero |
| | | $PO_4$ | 6.35 | 7.92 | 14.25 | 31.50 | |
| | | $NO_2$ | 5.86 | 2.78 | 13.16 | 44.67 | |
| | | $NO_3$ | 5.38 | 1.98 | 12.08 | 56.75 | |
| | | T | 5.02 | 5.21 | 11.26 | 68.01 | |
| Groups A & Ju | 19.80 | GLU | 5.40 | 1.82 | 27.27 | 27.27 | hetero |
| | | AP | 3.49 | 0.57 | 17.61 | 44.87 | |
| | | LAP | 2.51 | 0.62 | 12.66 | 57.54 | |
| | | TSM | 2.34 | 1.05 | 11.82 | 69.36 | |

**SIMPER on a spatial scale**

| | Average squared distance (D2) | Variable | Av.Sq. Dist | Sq.Dist/SD | Contrib% | Cum.% | Trophic pathway |
|---|---|---|---|---|---|---|---|
| Groups FC_E & K1_C | 27.23 | chla>10 | 4.57 | 0.83 | 16.78 | 16.78 | auto |
| | | chla_20_5 | 4.45 | 0.46 | 16.35 | 33.13 | |
| Groups FC_E & T2_C | 16.35 | TSM | 2.69 | 0.52 | 16.48 | 16.48 | auto |
| | | S | 2.38 | 0.78 | 14.59 | 31.07 | |
| | | chla_10_2 | 2.05 | 0.51 | 12.54 | 43.61 | |
| Groups FC_E & T3_C | 18.79 | TSM | 3.25 | 0.55 | 17.29 | 17.29 | auto |
| | | chla_10_2 | 3.02 | 0.48 | 16.09 | 33.39 | |
| | | DO | 2.59 | 0.61 | 13.76 | 47.15 | |
| | | T Chla | 2.52 | 0.74 | 13.42 | 60.56 | |
| Groups K1_C & T2_C | 14.56 | chla_20_5 | 3.05 | 0.45 | 20.95 | 20.95 | auto+hetero |
| | | AP | 2.26 | 0.50 | 15.52 | 36.47 | |
| | | chla>10 | 1.87 | 1.09 | 12.87 | 49.34 | |
| | | chla_10_2 | 1.84 | 0.46 | 12.63 | 61.98 | |
| Groups K1_C & T3_C | 15.12 | chla_20_5 | 3.34 | 0.45 | 22.10 | 22.10 | auto+hetero |
| | | chla_10_2 | 2.84 | 0.46 | 18.77 | 40.87 | |
| | | LAP | 2.26 | 0.68 | 14.93 | 55.80 | |
| Groups T2_C & T3_C | 4.67 | LAP | 1.10 | 0.49 | 23.64 | 23.64 | hetero |
| | | DO | 1.05 | 0.49 | 22.53 | 46.16 | |
