# Supplementary material for: Trophic and Microbial Dynamics in a Mediterranean Transitional Ecosystem (Lake Faro, Southern Italy): Implications for Pinna nobilis Conservation
Source: Microorganisms. 2026 Feb 11;14(2):423. doi: 10.3390/microorganisms14020423 (PMC12942930; doi:10.3390/microorganisms14020423)
Supplement: Supplementary file 1 [file microorganisms-14-00423-s001.zip › Supplementary Table S2.pdf]

Table S2. Pearsons' correlation coefficients computed per each station among biological and environmental parameters.

Stat K1_C

	T	S	DO	pH	LAP	AP	GLU	PO_4	NH_4	NO_3+NO_2	NO_2	NO_3	TSM	T Chl-a	chla pico	chla nano	chla micro	*P. nobilis*	PA
T	1.00																		
S	0.67	1.00																	
DO	-0.95*	-0.86	1.00																
pH	0.19	-0.58	0.10	1.00															
LAP	0.32	-0.06	-0.17	0.53	1.00														
AP	0.07	-0.59	0.17	0.79	-0.09	1.00													
GLU	-0.03	-0.62	0.25	0.73	-0.19	0.99**	1.00												
PO_4	-0.71	-0.10	0.51	-0.48	-0.25	-0.47	-0.38	1.00											
NH_4	-0.55	0.22	0.29	-0.91*	-0.68	-0.61	-0.51	0.71	1.00										
NO_3+NO_2	-0.93*	-0.47	0.84	-0.41	-0.25	-0.36	-0.27	0.66	0.66	1.00									
NO_2	-0.88	-0.27	0.71	-0.54	-0.34	-0.47	-0.37	0.92*	0.80	0.90*	1.00								
NO_3	-0.92*	-0.49	0.85	-0.38	-0.23	-0.33	-0.25	0.60	0.63	0.99**	0.86	1.00							
TSM	0.85	0.76	-0.90*	-0,16	-0.23	0.05	0.01	-0.56	-0.14	-0.79	-0.69	-0.79	1.00						
T Chl-a	0.12	-0.02	-0.04	-0,11	-0.35	0.19	0.19	-0.63	0.02	0.04	-0.30	0.10	0.32	1.00					
chla pico	0.15	0.01	-0.08	-0,11	-0.43	0.25	0.26	-0.64	0.03	-0.03	-0.34	0.03	0.39	0.99**	1.00				
chla nano	0.65	-0.07	-0.43	0.85	0.55	0.63	0.54	-0.63	-0.95*	-0.82	-0.82	-0.80	0.31	-0.17	-0.14	1.00			
chla micro	-0.22	-0.07	0,.23	-0.36	-0.22	-0.22	-0.21	-0.26	0.31	0.48	0.13	0.53	-0.07	0.84	0.78	-0.54	1.00		
P. nobilis	0.26	0.74	-0.49	-	-0.66	-0.58	-	0.86	0.93*	0.05	0.33	0.08	0.74	0.50	0.55	-	0.40	1.00	

				0.96**			0.52									0.74			
PA	-0.38	-0.84	0.60	0.76	0.58	0.43	0.41	0.03	-0.53	0.22	0.07	0.25	-0.75	-0.30	-0.35	0.37	-0.19	-0.97**	1.00

Stat. T2_C	T	S	DO	pH	LAP	AP	GLU	PO_4	NH_4	NO_3+NO_2	NO_2	NO_3	TSM	T Chl-a	chla pico	chla nano	chla micro	*P. nobilis*	PA
T	1.00																		
S	0.78	1.00																	
DO	-0.89*	-0.73	1.00																
pH	0.24	-0.30	-0,01	1.00															
LAP	0.01	-0.20	0.44	0.59	1.00														
AP	0.55	0.04	-0.32	0.94*	0.50	1.00													
GLU	0.01	-0.61	-0.04	0.66	0.11	0.51	1.00												
PO_4	-0.81	-0.33	0.57	-0.57	-0.41	-0.72	-0.42	1.00											
NH_4	-0.86	-0.60	0.54	-0.45	-0.50	-0.68	-0.01	0.89*	1.00										
NO_3+NO_2	-0.88*	-0.48	0.82	-0.57	0.01	-0.80	-0.31	0.76	0.72	1.00									
NO_2	-0.90*	-0.48	0.74	-0.42	-0.20	-0.62	-0.35	0.97**	0.86	0.81	1.00								
NO_3	-0.86	-0.47	0.81	-0.57	0.03	-0.80	-0.30	0.71	0.69	0.99**	0.77	1.00							
TSM	-0.27	-0.60	0.05	0.48	-0.27	0.33	0.68	0.18	0.44	-0.20	0.19	-0.24	1.00						
T Chl-a	0.13	0.25	0.21	-0.16	0.63	-0.12	-0.34	-0.33	-0.48	0.25	-0.25	0.30	-0.88	1.00					
chla pico	0.27	0.40	0.09	-0.12	0.63	-0.03	-0.41	-0.40	-0.59	0.11	-0.33	0.16	-0.91*	0.98*	1.00				
chla nano	0.01	0.11	0.33	-0.15	0.65	-0.16	-0.27	-0.27	-0.38	0.35	-0.17	0.40	-0.82	0.89*	0.95*	1.00			
chla micro	0.46	0.71	-0.43	-0.62	-0.15	-0.40	-	-0.27	-0.38	0.01	-0.39	0.05	-0.85	0.63	0.66	0.56	1.00		

	T	S	DO	pH	LAP	AP	GLU	PO_4	NH_4	NO_3+NO_2	NO_2	NO_3	TSM	T Chl-a	chla pico	chla nano	chla micro	*P. nobilis*	PA
							0.49												
P. nobilis	0.68	0.76	-0.85	-0.73	-0.89*	-0.51	-0.45	0.36	0.06	-0.26	-0.54	-0.25	-0.25	-0.24	-0.17	-0.32	0.70	1.00	
PA	-0.44	-0.73	0.63	0.74	0.61	0.50	0.46	0.06	0.12	0.08	0.26	0.06	0.54	-0.17	-0.21	-0.09	-0.86	-0.94*	1.00

Stat T3_C	T	S	DO	pH	LAP	AP	GLU	PO_4	NH_4	NO_3+NO_2	NO_2	NO_3	TSM	T Chl-a	chla pico	chla nano	chla micro	*P. nobilis*	PA
T	1.00																		
S	0.81	1.00																	
DO	-0.58	-0.25	1.00																
pH	0.20	-0.34	-0.47	1.00															
LAP	-0.10	-0.02	0.79	-0.03	1.00														
AP	0.27	-0.32	-0.31	0.75	0.09	1.00													
GLU	-0.14	-0.67	-0.33	0.65	-0.23	0.85	1.00												
PO_4	-0.73	-0.34	0.20	-0.55	-0.42	-0.69	-0.22	1.00											
NH_4	-0.72	-0.42	0.08	-0.44	-0.53	-0.57	-0.06	0.98**	1.00										
NO_3+NO_2	-0.94*	-0.57	0.59	-0.51	0.01	-0.54	-0.13	0.88*	0.84	1.00									
NO_2	-0.82	-0.41	0.41	-0.43	-0.13	-0.73	-0.32	0.92*	0.87*	0.91*	1.00								
NO_3	-0.94*	-0.59	0.61	-0.51	0.02	-0.51	-0.10	0.86	0.83	0.99**	0.89*	1.00							
TSM	-0.17	-0.59	-0.61	0.54	-0.67	0.54	0.86	0.13	0.30	-0.01	-0.05	0.01	1.00						
T Chl-a	0.16	0.16	0.61	-0.16	0.84	0.29	-0.07	-0.57	-0.64	-0.20	-0.47	-0.17	-0.56	1.00					
chla pico	0.53	0.14	0.01	0.40	0.50	0.78	0.37	-0.92*	-0.89*	-0.69	-0.89*	-0.66	-0.08	0.76	1.00				
chla nano	-0.06	0.14	0.82	-0.44	0.85	-0.04	-0.31	-0.26	-0.37	0.11	-0.14	0.13	-0.71	0.93*	0.47	1.00			

chla micro	0.32	-0.15	0.02	0.64	0.52	0.88	0.56	-0.87*	-0.81	-0.58	-0.76	-0.55	0.10	0.63	0.93*	0.33	1.00		
P. nobilis	0.12	0.59	0.47	-0.99**	0.22	-0.73	-0.65	0.82	0.31	0.44	0.37	0.43	-0.68	0.57	-0.17	0.68	-0.63	1.00	
PA	-0.42	-0.75	0.01	0.79	0.16	0.44	0.55	-0.09	-0.02	0.10	0.14	0.09	0.44	-0.20	0.03	-0.30	0.39	-0.89*	1.00

Stat. FC_E	T	S	DO	pH	LAP	AP	GLU	PO_4	NH_4	NO_3+NO_2	NO_2	NO_3	TSM	T Chl-a	chla pico	chla nano	chla micro	*P. nobilis*	PA
T	1.00																		
S	0.73	1.00																	
DO	-0.77	-0.47	1.00																
pH	0.18	0.48	0.44	1.00															
LAP	-0.26	-0.20	0.07	-0.36	1.00														
AP	-0.14	0.39	0.56	0.86	-0.42	1.00													
GLU	-0.26	0.33	0.46	0.63	-0.51	0.93*	1.00												
PO_4	-0.83	-0.92*	0.60	-0.32	-0.06	-0.09	0.04	1.00											
NH_4	-0.74	-0.88*	0.33	-0.57	-0.06	-0.30	-0.07	0.95*	1.00										
NO_3+NO_2	-0.88*	-0.88*	0.63	-0.29	-0.06	-0.02	0.13	0.99**	0.94*	1.00									
NO_2	-0.87*	-0.87*	0.42	-0.62	0.40	-0.35	-0.18	0.85	0.88*	0.86	1.00								
NO_3	-0.87*	-0.87*	0.64	-0.26	-0.05	0.01	0.15	0.99**	0.93*	0.99**	0.84	1.00							
TSM	-0.20	-0.78	0.24	-0.23	-0.03	-0.45	-0.54	0.61	0.53	0.52	0.39	0.53	1.00						
T Chl-a	-0.70	-0.59	0.96**	0.40	0.02	0.42	0.29	0.66	0.39	0.67	0.42	0.68	0.48	1.00					
chla pico	-0.49	-0.43	0.89*	0.53	0.13	0.41	0.17	0.42	0.11	0.41	0.20	0.43	0.46	0.94*	1.00				
chla nano	-0.68	-0.46	0.93*	0.49	-0.29	0.62	0.58	0.66	0.42	0.69	0.32	0.71	0.31	0.93*	0.80	1.00			

chla micro	-0.75	-0.84	0.22	-0.76	0.30	-0.49	-0.26	0.82	0.92*	0.82	0.97**	0.79	0.40	0.24	-0.01	0.17	1.00		
P. nobilis	-0.06	0.86	-0.46	-0.52	-0.22	-0.04	0.32	-0.09	0.94	0.08	0.28	0.05	-0.84	-0.65	-0.81	-0.36	0.60	1.00	
PA	-0.62	-0.48	0.94*	0.46	0.21	0.42	0.21	0.48	0.19	0.49	0.32	0.50	0.40	0.96*	0.98**	0.82	0.11	-0.73	1.00

*, p<0.05; **, p<0.01
